# Early therapeutic effects of an Angiopoietin-1 mimetic peptide in middle-aged rats with vascular dementia

**DOI:** 10.3389/fnagi.2023.1180913

**Published:** 2023-05-25

**Authors:** Huanjia Gao, Elizabeth L. Findeis, Lauren Culmone, Brianna Powell, Julie Landschoot-Ward, Alex Zacharek, Trueman Wu, Mei Lu, Michael Chopp, Poornima Venkat

**Affiliations:** ^1^Department of Neurology, Henry Ford Health, Detroit, MI, United States; ^2^Public Health Sciences, Henry Ford Health, Detroit, MI, United States; ^3^Department of Physics, Oakland University, Rochester, MI, United States; ^4^Department of Physiology, Michigan State University, East Lansing, MI, United States

**Keywords:** Angiopoietin-1, cognition, microinfarct dementia, vascular dementia (VaD), Vasculotide, glymphatic clearance function, AV-001

## Abstract

**Background:**

Vascular Dementia (VaD) refers to dementia caused by cerebrovascular disease and/or reduced blood flow to the brain and is the second most common form of dementia after Alzheimer’s disease. We previously found that in middle-aged rats subjected to a multiple microinfarction (MMI) model of VaD, treatment with AV-001, a Tie2 receptor agonist, significantly improves short-term memory, long-term memory, as well as improves preference for social novelty compared to control MMI rats. In this study, we tested the early therapeutic effects of AV-001 on inflammation and glymphatic function in rats subjected to VaD.

**Methods:**

Male, middle-aged Wistar rats (10–12 m), subjected to MMI, were randomly assigned to MMI and MMI + AV-001 treatment groups. A sham group was included as reference group. MMI was induced by injecting 800 ± 200, 70–100 μm sized, cholesterol crystals into the internal carotid artery. Animals were treated with AV-001 (1 μg/Kg, i.p.) once daily starting at 24 h after MMI. At 14 days after MMI, inflammatory factor expression was evaluated in cerebrospinal fluid (CSF) and brain. Immunostaining was used to evaluate white matter integrity, perivascular space (PVS) and perivascular Aquaporin-4 (AQP4) expression in the brain. An additional set of rats were prepared to test glymphatic function. At 14 days after MMI, 50 μL of 1% Tetramethylrhodamine (3 kD) and FITC conjugated dextran (500 kD) at 1:1 ratio were injected into the CSF. Rats (4–6/group/time point) were sacrificed at 30 min, 3 h, and 6 h from the start of tracer infusion, and brain coronal sections were imaged using a Laser scanning confocal microscope to evaluate tracer intensities in the brain.

**Result:**

Treatment of MMI with AV-001 significantly improves white matter integrity in the corpus callosum at 14 days after MMI. MMI induces significant dilation of the PVS, reduces AQP4 expression and impairs glymphatic function compared to Sham rats. AV-001 treatment significantly reduces PVS, increases perivascular AQP4 expression and improves glymphatic function compared to MMI rats. MMI significantly increases, while AV-001 significantly decreases the expression of inflammatory factors (tumor necrosis factor-α (TNF-α), chemokine ligand 9) and anti-angiogenic factors (endostatin, plasminogen activator inhibitor-1, P-selectin) in CSF. MMI significantly increases, while AV-001 significantly reduces brain tissue expression of endostatin, thrombin, TNF-α, PAI-1, CXCL9, and interleukin-6 (IL-6).

**Conclusion:**

AV-001 treatment of MMI significantly reduces PVS dilation and increases perivascular AQP4 expression which may contribute to improved glymphatic function compared to MMI rats. AV-001 treatment significantly reduces inflammatory factor expression in the CSF and brain which may contribute to AV-001 treatment induced improvement in white matter integrity and cognitive function.

## Introduction

Vascular Dementia (VaD) refers to cognitive dysfunction and neurological impairment caused by cerebrovascular disease and/or reduced cerebral blood flow and subsequent infarctions in the brain. VaD alone or in combination with Alzheimer’s disease (AD) as mixed dementia remains the second most common cause of dementia, and accounts for more than 20% of all dementia cases (Khan et al., 2016; Mossanen Parsi et al., 2021). VaD patients often exhibit attention-deficit disorders, memory loss, slowed thinking, depression, confusion and disorientation, gait or balance problems, speech impairment, and loss of executive functions (Venkat et al., 2015). Thus, VaD affects the ability of patients to live and function independently and creates a huge socio-economic burden (Wimo et al., 2013). The risk of developing VaD is high in individuals with hypertension, obesity, diabetes, stroke, and cardiac disease, and this risk increases exponentially with advancing age (Gorelick et al., 2011; Zhao et al., 2020). Therefore, as life expectancy increases and the global aging populations grows, there is a pressing need to develop treatments specifically for VaD. Neuropathological diagnosis of VaD relies largely on the presence of ischemic or hemorrhagic infarcts due to stroke, or commonly lacunar infarcts due to the occlusions of deep penetrating arteries, and microinfarcts due to aging and vascular pathologies (Corrada et al., 2016; Iadecola et al., 2019; Oveisgharan et al., 2022). We have previously reported that treatment of middle-aged rats subjected to a multiple microinfarct (MMI) model of VaD with AV-001 significantly improves short-term and long-term memory, preference for social novelty, spatial learning and memory at 6 weeks after MMI (Culmone et al., 2022). AV-001 treatment significantly reduced demyelination, improved axon density, and neuroplasticity in middle-aged rats with VaD (Culmone et al., 2022). However, the mechanisms by which AV-001 improves cognitive function in rats with VaD are unclear. This study investigates the early therapeutic benefits of AV-001 in middle-aged rats with VaD.

AV-001 is a novel Tie2 receptor agonist designed to activate the angiopoietin/Tie2 signaling pathway (Gutbier et al., 2017; Dekker et al., 2018). AV-001 is a synthetic PEGylated peptide conjugate derived from 4 identical 7-amino-acid peptides (T7) bound to the PEG tetramer. The T7 peptide, which forms the core of AV 001, was selected from over a billion unique peptide sequences using a phage-display method. The peptide was chosen for its ability to bind to the extracellular region of the Tie2 receptor (Tournaire et al., 2004). AV-001 is a clinical candidate version of its predecessor analog referred to as Vasculotide. Vasculotide has been demonstrated to bind and phosphorylate Tie2 in a dose-dependent manner (Gutbier et al., 2017; Dekker et al., 2018). The role of Angiopoietin-1 (Angpt-1) in post-ischemic recovery is well documented and Angpt-1 is known to promote vascular remodeling via pericyte recruitment, and maturation, and stabilization of blood vessels (Suri et al., 1996, 1998; Iurlaro et al., 2003), and to promote neurite remodeling (Wang et al., 2015), and attenuate blood vessel leakage in the ischemic brain (Zhang et al., 2002; Metheny-Barlow et al., 2004). In diabetic rats subjected to ischemic stroke, Vasculotide treatment significantly improves stroke outcome and neurological function by reducing neuroinflammation and blood brain barrier leakage in the ischemic penumbra (Venkat et al., 2018, 2021). The safety and efficacy of AV-001 in improving disease outcome has been well documented using several rodent disease models including sepsis (Kümpers et al., 2011), influenza (Sugiyama et al., 2015), stroke (Venkat et al., 2018, 2021), VaD (Culmone et al., 2022), and AD (Lynch et al., 2021). In our prior study, we found that 1 μg/Kg AV-001 administered once daily starting at 24 h after MMI does not alter body weight, blood pressure, or heart rate at 6 weeks after MMI compared to baseline measurements or MMI control rats (Culmone et al., 2022).

Cerebral small vessel disease (cSVD) is one of the most common causes of VaD that is characterized by perivascular space (PVS) dilation, white matter hyperintensities and lacunar infarcts (Benveniste and Nedergaard, 2022). PVS serves as the main conduit for lymphatics drainage (Da Mesquita et al., 2018). Enlarged PVS likely reflects dysfunction of glymphatic system, which may further lead to the aggregation of hazardous wastes, potentially damaging the brain (Brown et al., 2018). In our prior studies, we reported that animals subjected to MMI exhibit reduced cerebral blood flow, multiple diffuse cerebral microinfarcts, white matter injury, impairment of glymphatic waste clearance pathway, and cognitive impairment (Venkat et al., 2017; Yu et al., 2019a). The glymphatic system is a molecular size selective mechanisms of waste clearance that enables removal of soluble proteins, metabolic wastes and neurotoxins such as soluble Aβ, tau and lactate from the brain as well as facilitates the delivery of signaling molecules and metabolic factors to the brain (Yu et al., 2022; Iadecola et al., n.d.). The glymphatic pathway consists of influx of cerebrospinal fluid (CSF) along paraarterial spaces, subsequent transport of CSF into the brain interstitium, facilitated by aquaporin 4 (AQP4) lined water channels, CSF exchange with interstitial fluid followed by paravenous interstitial fluid efflux (Iliff et al., 2012; Benveniste et al., 2019). In diabetic and hypertensive rodent models of cSVD, impairment of glymphatic function with stagnation of glymphatic flux and perivascular dilation occur as SVD progresses and accelerates accumulation of protein aggregates and metabolic (Benveniste and Nedergaard, 2022). In hypertension, diabetes, and aging, arterial wall stiffening affecting arterial wall pulsatility, and/or inflammation causes a reduction in CSF influx into the brain parenchyma, and stagnation of perivascular fluid leads to PVS dilation (Benveniste and Nedergaard, 2022). Impaired glymphatic function likely precedes cognitive decline in diabetes (Jiang et al., 2017), as well as MMI induced VaD (Venkat et al., 2017; Wang et al., 2017; Yu et al., 2019a). Therefore, here, we test whether AV-001 treatment improves white matter integrity, reduces inflammation, and improves glymphatic waste clearance at 14 days after MMI in middle-aged rats.

## Methods

The research procedures were in compliance with the National Institutes of Health (NIH) Guide for the Care and Use of Laboratory Animals and were approved by the Institutional Animal Care and Use Committee (IACUC) of the Henry Ford Health System. This manuscript was prepared following the ARRIVE guidelines (Kilkenny et al., 2010).

### MMI model, AV-001 treatment and experimental groups

Male, Wistar rats were obtained as retired breeders from Charles River Laboratories (Wilmington, MA), housed 2 per cage with access to food and water *ad libitum*, and aged to 10 months. Cholesterol crystals were prepared following previously described methods (Rapp et al., 2008a; Venkat et al., 2017; Wang et al., 2017; Yu et al., 2019a; Chandran et al., 2020). In brief, freshly prepared cholesterol crystals were filtered using 100 and 70 μm cell strainers and counted with a hemocytometer. A final concentration of 800 ± 100 crystals/300 μL saline was prepared and loaded into a 1 cc syringe attached to a PE-50 tube with a tapered tip. Rats were anesthetized using 4% isoflurane and then spontaneously respired with 2% isoflurane mixed with 2:1 N2O:O2 using a nose cone. Anesthesia was regulated using a modified FLUOTEC 3 Vaporizer (Fraser Harlake) and the animals were placed on a heating pad maintained at 37°C throughout the surgery. Rats were placed in a supine position and a ~1.5 cm small central midline incision was made on the neck. Under a dissecting microscope, the carotid bifurcation was exposed and while carefully avoiding any damage to the muscles or vagus nerve, the CCA and ICA were isolated and temporarily clamped. The distal end of the ECA was permanently occluded using a 4–0 silk suture. The catheter loaded with cholesterol crystal was gently advanced into the lumen of the ICA via an incision on the ECA and cholesterol crystals were slowly injected into the ICA, while the CCA remained clamped. The catheter was gently removed, and the ECA was ligated while the CCA and ICA remained patent. The neck incision was closed with a 4–0 nylon suture. Animals received routine post-surgical support and care including analgesia (Buprenorphine SR, 1 mg/Kg, subcutaneously). Stock solutions and aliquots of AV-001 were prepared in Dulbecco’s phosphate-buffered saline and stored at −20°C. AV-001 treatment was initiated at 24 h after MMI and administered via i.p. injection once daily for 14 days until sacrifice. Out of 18 rats subjected to MMI, 3 rats died within day 3. Thus, for immunohistochemical evaluation, CSF cytokine array and brain RT-PCR, the following sample sizes were employed: (1) Sham (*n* = 13); (2) MMI (*n* = 8); (3) MMI + 1 μg/Kg AV-001 (*n* = 7).

### Euthanasia

Rats were sacrificed at 14 days after MMI. Rats were anesthetized using Ketamine (87 mg/Kg) and Xylazine (13 mg/Kg). The head was fixed using a stereotaxic apparatus at a 45° angle between animal’s head and horizontal line. A median incision from the forehead to the neck was used to expose the base of the skull. A 27G needle was inserted gently into the cisterna magna and approximately 0.1 mL of uncontaminated CSF was collected and stored at −80°C. Following transcardial perfusion with 0.9% saline, the brains were quickly removed. A small section of cortical tissue was flash frozen in liquid nitrogen and stored at −80°C while the rest of the brain was immersion fixed in 4% paraformaldehyde.

### Histological and immunohistochemical assessment

Paraffin embedded brain coronal tissue sections were prepared, and Hematoxylin and eosin (H&E) staining was used to assess white matter integrity. Three fields of view encompassing both medial and lateral corpus callosum were captured for each rat, and white matter damage was graded on a scale of 0 to 3. An investigator blinded to the groups rated the corpus callosum as normal (grade 0), showing disarrangement of nerve fibers (grade 1), having marked vacuole formation (grade 2), or having regions with the disappearance of myelinated fibers (grade 3; Wakita et al., 1994; Shibata et al., 2004). Antibodies against myelin basic protein (MBP, 1:250, Millipore) and AQP4 (1:1,500, Millipore) were also used. To evaluate PVS and water channel dysfunction, 6–8 fields of view of cortex and striatum with blood vessels were digitized under a 20× objective (Olympus BX40) using a 3-CCD color video camera with an MCID image analysis system (Imaging Research). For each field of view, all large blood vessels in the cortex and striatum with diameter > 10 μm were selected and PVS was quantified by manually drawing outer and inner limits of each blood vessel and calculating the differential area in μm^2^ following previously described methods (Ampawong et al., 2011; Venkat et al., 2017). For MBP and AQP4, positive-stained areas were measured using an in-built densitometry function with a density threshold set uniformly above unstained for all groups. The data for each animal were averaged to determine the percentage of positive area.

### CSF cytokine array

To evaluate early inflammatory factor expression in CSF, a cytokine array was employed (R&D Systems) and CSF samples were pooled by group and tested in duplicates (Sham, MMI and MMI + 1 μg/Kg). Protein concentration was determined with the BCA kit (Thermo Scientific) and CSF volume containing 150 μg protein was used for each group. The immunoblot images were acquired using a Biotechne fluorChem E system and images were analyzed using ImageJ software.

### Real time polymerase chain reaction assay

The standard TRIzol (Invitrogen) protocol was used to isolate total RNA. Subsequently, the M-MLV standard protocol was used to convert 2 μg of Total RNA into cDNA (complementary DNA). A 2 μL aliquot of the resulting cDNA was used to conduct quantitative polymerase chain reaction (qPCR) using the SYBR Green real-time PCR method. The qPCR was performed on a ViiA 7 PCR instrument (Applied Biosystems) with program parameters recommended by the manufacturer, including a 2-min incubation at 50°C, a 10-min incubation at 95°C, followed by 40 cycles of amplification: 15 s at 95°C and 1 min at 60°C. Each sample was tested in triplicate, and gene expression analysis was performed using the 2^−ΔΔCT^ method to determine relative gene expression levels.

Endostatin:

F: GGAGGCTGATGGAGAGTTACTG; R: ACAGGACGATGTAGCTGTTGTG.

Thrombin R:

F: AGTCCCTGTCCTGGCGCACT; R: GGACGTCGTGGCAGGTGGTG.

TNF-α:

F: TACTCCCAGGTTCTCTTCAAGG; R: GGAGGTTGACTTTCTCCTGGTA.

IL-6:

F: CAGAGTGTGGGCGAACAAAG; R: CAGCCTTAGCAAAAACTCTCTGG.

PAI-1:

F: GGGCAGCAGATAGACAGATC G; R: CTGAA ATAACACAAGGCGGC.

CXCL-9:

F: TCCCCTAGACGGTTGTGGATG; R: TACTCTGAAATGCTGAGCGGC.

### Glymphatic function assessment

To evaluate glymphatic function, additional sets of middle-aged (10–12 months) male rats were randomized to Sham, MMI and MMI + 1 μg/Kg AV-001 groups. AV-001 treatment was initiated 24 h after MMI and administered via i.p. injection once daily for 14 days. At 14 days after MMI, animals were anesthetized with 4% isoflurane in a chamber and then spontaneously respired with 2% isoflurane mixed within 2:1 N2O:O2 mixture using a nose cone. Anesthesia was regulated using a modified FLUOTEC 3 Vaporizer (Fraser Harlake) and the animals were placed on a heating pad maintained at 37°C throughout the surgery. The head was fixed using a stereotaxic apparatus. Following a midline dorsal neck incision to expose the atlanto-occipital membrane, a hole was drilled at the lower edge of the occiput, and a PE50 tube with a tapered tip was gently inserted into the cisterna magna for about 1.5–2 mm. The external portion of the catheter was affixed to the occipital bone using superglue. Then, 50 μL of 1% (diluted in artificial CSF) Tetramethylrhodamine conjugated dextran (MW: 3 kD, Thermo Fisher) and FITC conjugated dextran (MW: 500 kD, Thermo Fisher) at 1:1 ratio were injected into the CSF over 25 min using a syringe pump. Rats (4–6/group/time point) were sacrificed at 30 min, 3 h, and 6 h from the start of infusion and perfusion fixed with 0.9% saline followed by 4% Paraformaldehyde. Vibratome sections (100 μm thick) were cut to analyze tracer movement along paravascular pathways. A laser scanning confocal microscope (Olympus FV1200) equipped with a motorized stage was used. To obtain whole slice montages, the brain coronal sections were imaged under a 10× objective in the x-y direction using tile scans that were stitched into larger mosaics. Four optical sections were acquired along the z-axis (depth) and a single composite projection image was constructed. All images acquired from laser scanning confocal microscope were imported into an MCID image analysis system and an in-built densitometry function with a density threshold set uniformly above unstained was used to evaluate the percentage of brain section that was fluorescently marked, i.e., tracer intensity in brain was calculated.

### Statistical analysis

Data are presented as mean ± SEM for illustration. Data were evaluated for normality and homoscedasticity; log transformation for PVS Cortex and PVS Striatum were considered to make data normally distributed. One-way Analysis of variance (ANOVA) was used to study the group (either MMI or AV-001) effect. Similarly, for glymphatic function measurements, repeated analysis of variance (ANCOVA) was used to study group (either MMI or AV-001) and the time effect. Statistical significance was detected at *p* < 0.05. A significant group by time interaction indicated that the group effects varied over time.

## Results

### AV-001 treatment significantly improves white matter integrity in the corpus callosum when compared to control MMI rats

**Figure 1:**
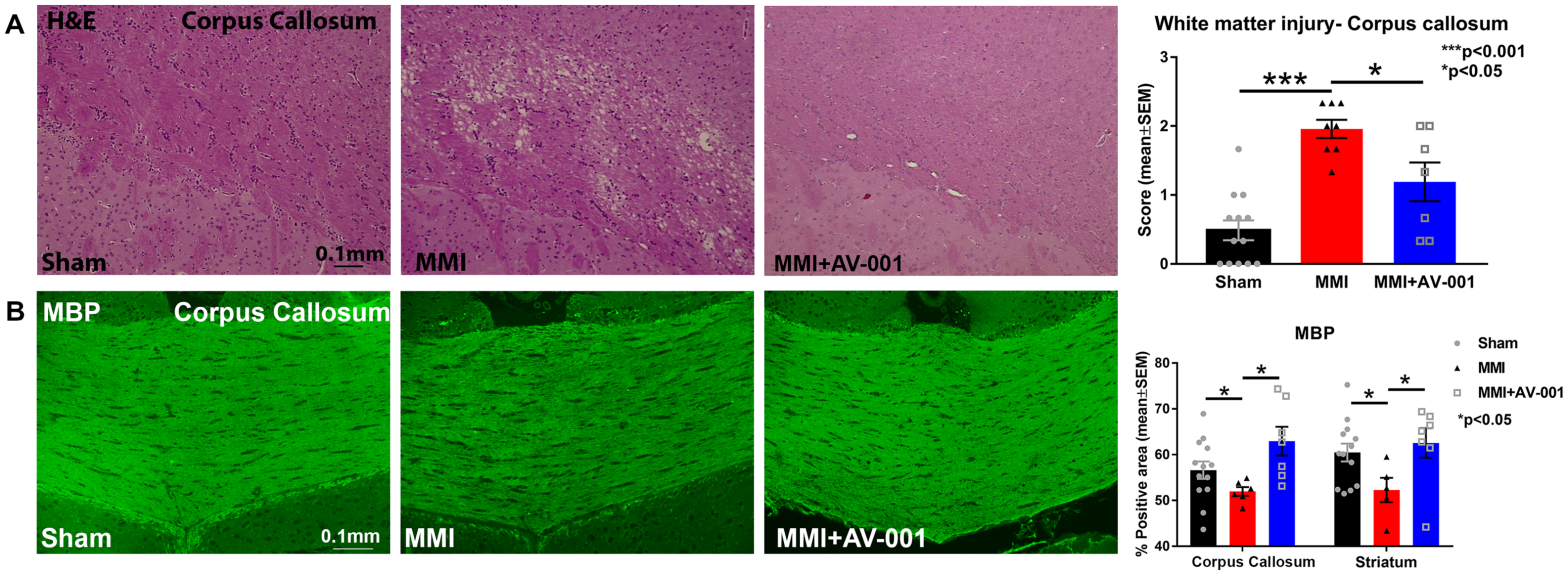
**(A)** H&E staining was used to evaluate white matter injury in the corpus callosum. Three fields of view, encompassing both medial and lateral corpus callosum, were captured for each rat, and white matter damage was graded on a scale of 0 to 3. The scores from the 3 fields of view were averaged to obtain a single score. The scores represented normal appearance (grade 0), disarrangement of the nerve fibers (grade 1), marked the formation of marked vacuoles (grade 2), or the disappearance of myelinated fibers (grade 3). MMI induces significant white matter injury in the corpus callosum compared to Sham rats at 14  days after MMI (*p* < 0.001). Treatment with 1 μg/Kg AV-001 significantly improves white matter integrity, indicated by reduced rarefaction and vacuolation in the corpus callosum when compared to control MMI rats (*p* < 0.05). **(B)** MBP staining indicates that MMI induces significant demyelination in the corpus callosum, and white matter bundles of the striatum compared to Sham rats at 14 days after MMI. Treatment with 1 μg/Kg AV-001 significantly attenuates demyelination compared to control MMI rats. Scale bar: 0.1 mm. Sham n = 13; MMI n = 8; MMI + AV-001 *n* = 7.

To test whether AV-001 treatment improves white matter integrity, H&E staining was employed, and white matter injury was scored on a scale of 0–3 and averaged over three fields of view encompassing both medial and lateral corpus callosum. The scores represented normal appearance (grade 0), disarrangement of the nerve fibers (grade 1), marked formation of vacuoles (grade 2), or the disappearance of myelinated fibers (grade 3; Wakita et al., 1994; Shibata et al., 2004). Our data in Figure 1A show that MMI induces significant white matter injury in the corpus callosum compared to Sham rats at 14 days after MMI. Treatment with 1 μg/Kg AV-001 significantly improves white matter integrity indicated by reduced rarefaction and vacuolation in the corpus callosum when compared to control MMI rats. Figure 1B shows that MMI induces significant demyelination in the corpus callosum and white matter bundles of the striatum compared to Sham rats at 14 days after MMI. Treatment with 1 μg/Kg AV-001 significantly attenuates demyelination compared to control MMI rats.

### AV-001 treatment significantly reduces PVS and increases perivascular AQP4 expression compared to MMI rats

**Figure 2:**
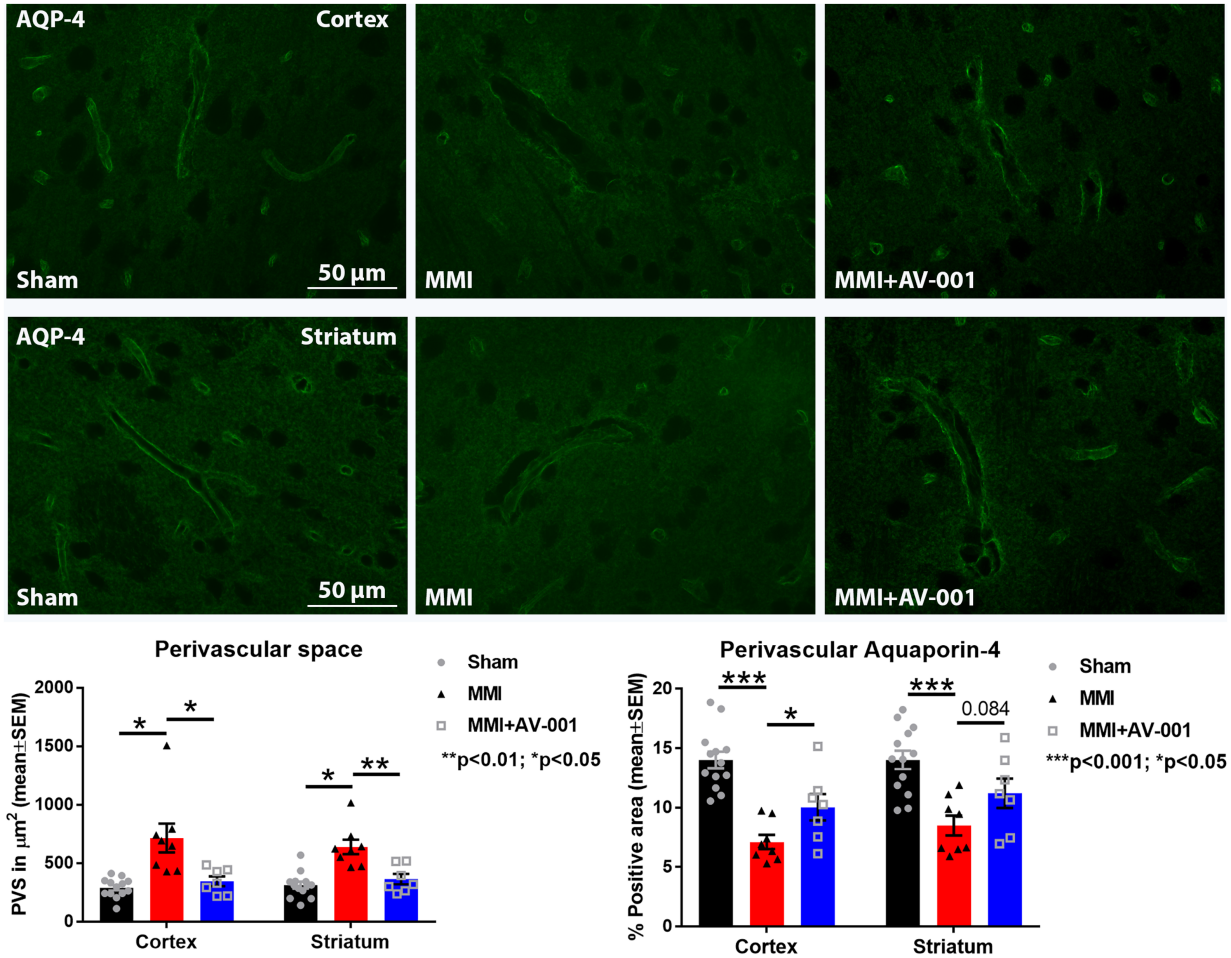
MMI induces significant dilation of the PVS in the cortex (*p* < 0.05) and striatum (*p* < 0.05) and reduces perivascular AQP4 expression in the cortex (*p* < 0.001) and striatum (*p* < 0.001) compared to Sham rats. Treatment of MMI with 1 μg/Kg AV-001 significantly reduces PVS dilation in the cortex (*p* < 0.05) and striatum (*p* < 0.01) and increases perivascular AQP4 expression in the cortex (*p* < 0.05) and striatum (*p* = 0.084) compared to MMI rats. Scale bar: 50 μm. Sham *n* = 13; MMI *n* = 8; MMI + AV-001 *n* = 7.

PVS, also known as Virchow-Robin spaces, are fluid filled compartments surrounding the small blood vessels in the brain. PVS dilation is a hallmark of cerebral small vessel disease and dementia (Bown et al., 2022). AQP4 is a water channel protein that is expressed in astrocytes and ependymal cells lining the ventricles with the highest expression on perivascular astrocytic end-feet (Mader and Brimberg, 2019; Salman et al., 2022). Astrocytic end-feet are in close contact with cerebral vessels and have high perivascular coverage such that only ~20-nm clefts between overlapping end-feet provide access to the brain parenchyma. The particularly high AQP4 expression at the blood brain barrier and blood-CSF barrier facilitates bidirectional fluid exchange in the brain (Mader and Brimberg, 2019). In our prior work, we found that MMI induces PVS enlargement and reduces perivascular AQP4 expression which may contribute brain-wide glymphatic dysfunction (Venkat et al., 2017; Yu et al., 2019a). Hence, we have evaluated PVS dilation and perivascular AQP4 expression in this study. We found that AV-001 treatment of MMI significantly decreases the dilation of PVS and increases perivascular AQP4 expression compared to control MMI rats (Figure 2).

### AV-001 treatment significantly improves glymphatic function in rats subjected to an MMI model of VaD

**Figure 3:**
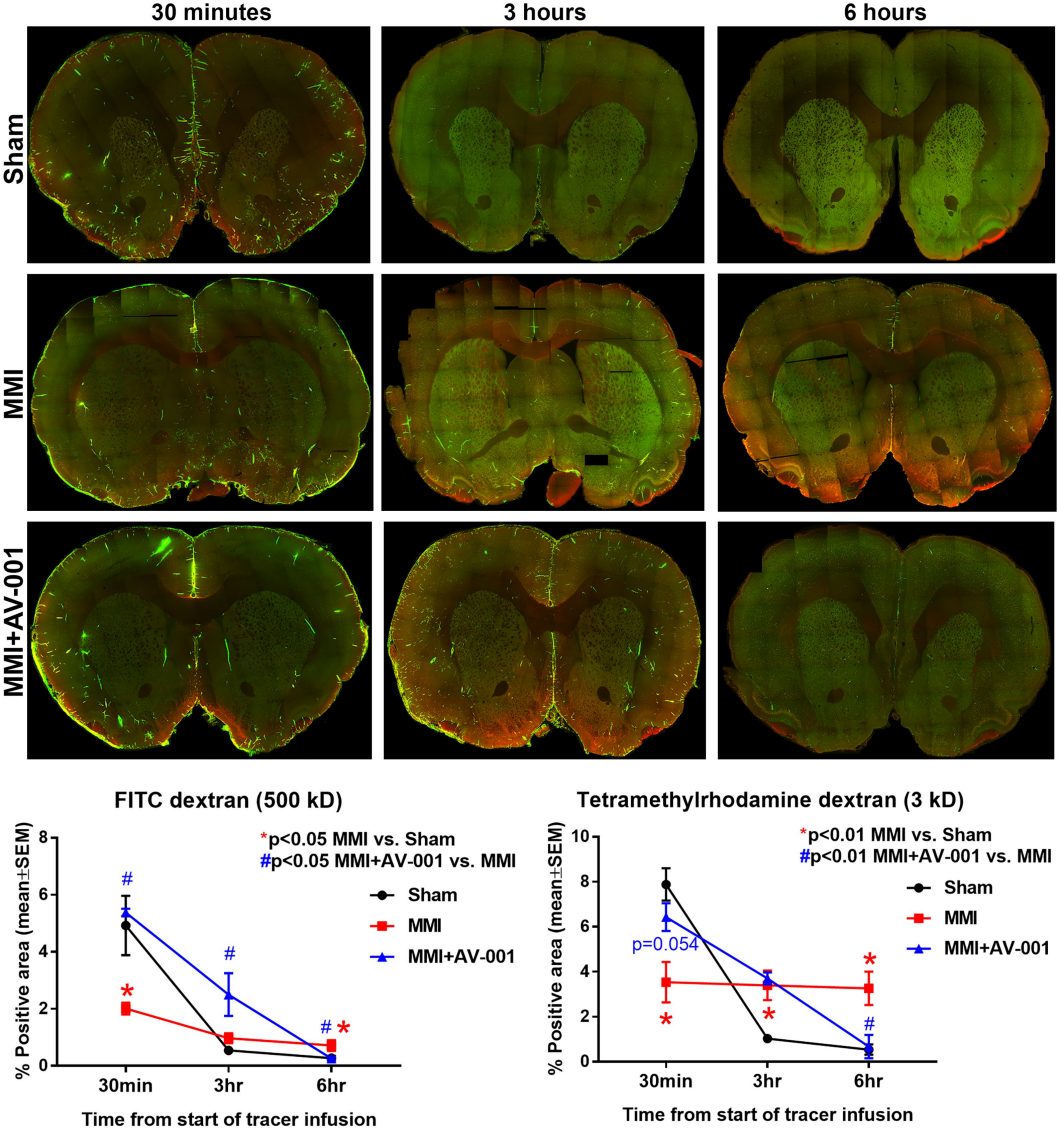
To evaluate glymphatic function at 14 days after MMI, fluorescent tracers (FITC-dextran and Tetramethylrhodamine dextran) were slowly injected into the cisterna magna and animals (*n* = 4–6/time point/group) were sacrificed at 30 min, 3 or 6 h after injection. Whole brain slice montages of 100 μm thick vibratome sections were obtained using a 10× objective (with 4 z-stacks) of a laser scanning confocal microscope. Tracer fluorescence intensities were quantified using an in-built densitometry function in MCID image analysis system. MMI induces significant glymphatic dysfunction with significantly (*p* < 0.05) reduced expression of both tracers at 30 min and significantly (*p* < 0.05) delayed clearance of both tracers at 6 h compared to Sham rats. 1 μg/Kg AV-001 treatment significantly (*p* < 0.05) improves glymphatic function indicated by increased expression of FITC dextran (*p* < 0.05) at 30 min and 3 h after infusion and increased expression of Tetramethylrhodamine dextran (*p* = 0.054) at 30 min after infusion, as well as significantly (*p* < 0.05) increased clearance of both tracers by 6 h after infusion when compared to MMI rats.

Impaired glymphatic function has been implicated to induce white matter damage and cognitive decline in rats subject to MMI, stroke, diabetes, and other neurological (Jiang et al., 2017; Venkat et al., 2017; Wang et al., 2017; Yu et al., 2019a; Lin et al., 2020). Thus, the glymphatic system represents an important target for therapeutic intervention in neurological diseases. To evaluate glymphatic function, two fluorescent tracers varying in molecular size were injected into the CSF and the progressive transport of intracisterna-injected tracers was analyzed by sacrificing rats at various time points following infusion (30 min, 3 and 6 h). Since the glymphatic system is a brain-wide waste clearance pathway, we have evaluated the intensities of injected tracers in whole-brain sections. Consistent with prior findings (Iliff et al., 2012; Jiang et al., 2017), we also observed the entry of both tracers into the paravascular spaces following injection into the cisterna magna of Sham rats. Over time, the Tetramethylrhodamine conjugated dextran which has a molecular size of 3 kD was able to readily enter the interstitium, while the larger FITC conjugated dextran, with a molecular size of 500 kD, was confined to paravascular space. Our data in Figure 3 show that MMI induces glymphatic dysfunction with significantly (*p* < 0.05) reduced expression of both tracers at 30 min and significantly (*p* < 0.05) delayed clearance of both tracers at 6 h compared to Sham rats. 1 μg/Kg AV-001 treatment significantly (*p* < 0.05) improves glymphatic function indicated by increased expression of FITC dextran (*p* < 0.05) at 30 min and 3 h after infusion as well as Tetramethylrhodamine dextran (*p* = 0.054) at 30 min after infusion; as well as significantly (*p* < 0.05) increased clearance of both tracers by 6 h after infusion when compared to MMI rats.

### MMI significantly increases, while AV-001 treatment significantly decreases the expression of inflammatory factors such TNF-α, CXCL9, and anti-angiogenic factors such as endostatin, PAI-1 and P-selectin in the CSF

**Figure 4:**
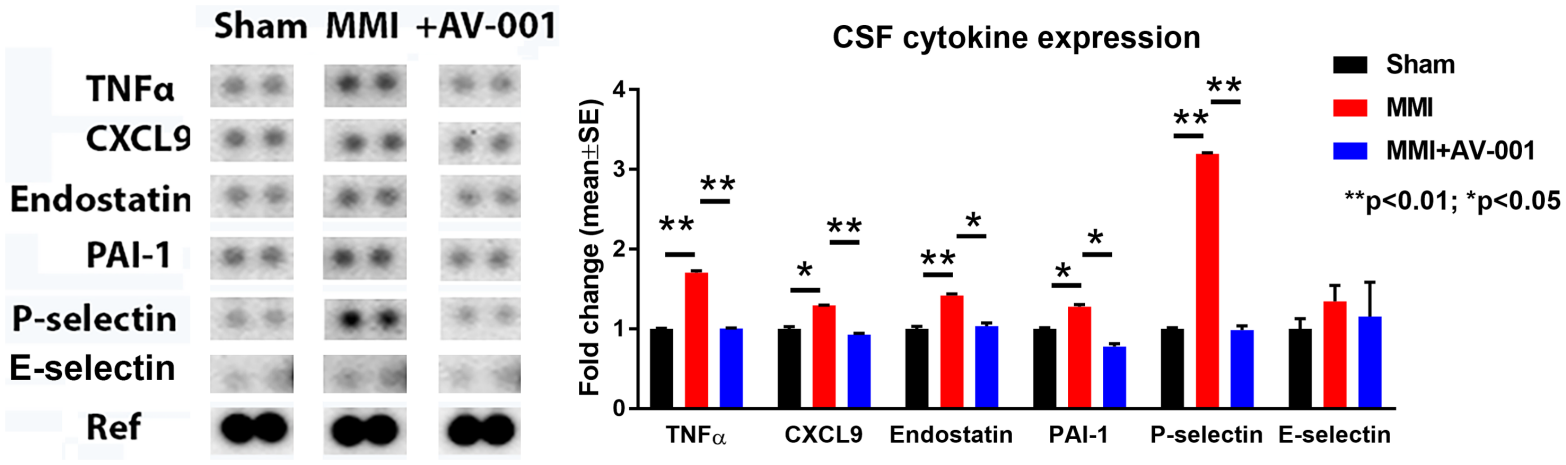
A multiplex antibody array kit was used to simultaneously evaluate the relative expression levels of several different cytokines, chemokines, and acute-phase proteins in the CSF. MMI significantly increases, while 1 μg/Kg AV-001 treatment significantly decreases the expression of inflammatory factors such TNF-α, CXCL9, and anti-angiogenic factors such as endostatin, PAI-1, and P-selectin in the CSF. CSF samples were pooled for each group. ^**^p < 0.01, *p < 0.05.

Early and sustained inflammation in the brain is a known contributor for white matter injury (Rosenberg, 2009; Ben-Ari et al., 2019). CSF cytokine array was used to evaluate the effects of AV-001 on inflammation after MMI. Data in Figure 4 show that MMI significantly increases, while AV-001 treatment significantly decreases the expression of inflammatory factors such as TNF-α, chemokine ligand 9 (CXCL9), and anti-angiogenic factors such as endostatin, plasminogen activator inhibitor-1 (PAI-1) and P-selectin.

### MMI significantly increases, while AV-001 treatment significantly reduces brain tissue expression of endostatin, thrombin, TNF-α, PAI-1, CXCL9, and IL-6

**Figure 5:**
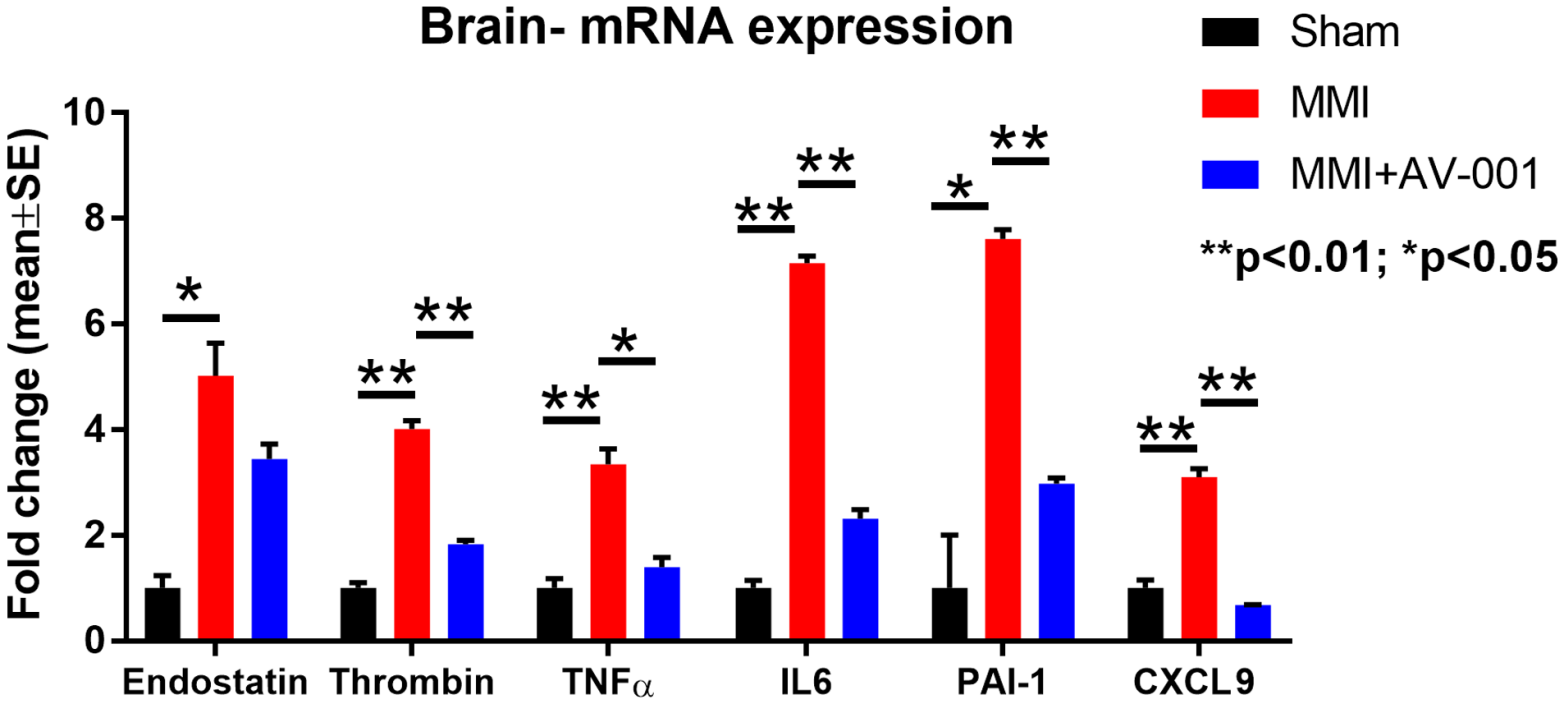
RT-PCR was used to evaluate the expression of inflammatory factors in the brain tissue. MMI significantly increases, while 1 μg/Kg AV-001 treatment significantly reduces brain tissue expression of endostatin, thrombin, TNF-α, and IL-6. Brain tissue samples were pooled for each group. ^**^p < 0.01, ^*^p < 0.05.

The results described above showed that AV-001 treatment dramatically decreases the expression of inflammatory factors (TNF-α, CXCL9), and anti-angiogenic factors such as endostatin, PAI-1 and P-selectin. We then performed real time polymerase chain reaction assay (RT-PCR) to test whether AV-001 treatment also decreases mRNA level of these inflammatory mediators and anti-angiogenic factors in brain tissue in order to further validate the results obtained with CSF cytokine array. Data in Figure 5 indicate that MMI increases, while AV-001 treatment significantly reduces brain tissue expression of endostatin, thrombin, TNF-α, IL-6, PAI-1 and CXCL9.

## Discussion

In this study, we have demonstrated that AV-001 treatment significantly improves white matter integrity, reduces PVS, and increases AQP4 expression which in-concert may contribute to improved glymphatic function in middle-aged rats subjected to this MMI model of VaD. We also report that AV-001 treatment significantly reduces inflammatory factor expression in the CSF and brain which also may contribute to the therapeutic effects of AV-001 in MMI induced VaD. These findings, in combination with our previous finding that AV-001 improves cognitive function and memory in middle-aged rats subjected to MMI (Culmone et al., 2022), provides evidence that AV-001 may be a novel therapeutic agent for the treatment of multi-infarct dementia.

AV-001 is a synthetic Angiopoietin-1 mimetic and a clinical candidate version of Vasculotide, previously reported in a model of ischemic stroke in diabetic rats to significantly improve stroke outcome and neurological function by reducing neuroinflammation and blood brain barrier leakage in the ischemic penumbra (Venkat et al., 2018, 2021). We also previously found that treatment of MMI with once daily administration of 1 μg/Kg AV-001 significantly enhances short-term and long-term memory, improves the preference for social novelty, and increases spatial learning and memory, as well as increases axon density, remyelination and neuroplasticity in the brain of middle-aged rats subjected to MMI (Culmone et al., 2022). Patients with VaD exhibit alterations in the microvasculature that supplies the subcortical white matter. These changes result in significant and extensive injury to the white matter including vacuolization, rarefaction, axonal loss, and demyelination (Iadecola, 2013). In our previous study, we showed that 1 μg/Kg AV-001 significantly reduces white matter injury in middle aged-rats subjected to this MMI model of VaD 6 weeks after MMI (Culmone et al., 2022). In this study, we evaluated white matter injury at 14 days after MMI and showed that MMI induces significant white matter injury in the corpus callosum compared to Sham rats, and that AV-001 treated rats exhibit significantly improved white matter integrity indicated by reduced rarefaction and vacuolation in the corpus callosum when compared to VaD control rats.

PVS dilation is a frequent finding in the pathology of cerebral small disease, and has been observed in animals subjected to the MMI model of VaD (Venkat et al., 2017; Wang et al., 2017; Kalaria, 2018; Venkat et al., 2020). Neuroimaging and pathological studies have demonstrated that dilated PVS increases with age and contributes to cognitive impairment (Patankar et al., 2005; Cumurciuc et al., 2006). A cohort study including more than 1,000 participants reported that a greater burden of PVS enlargement was linked to a higher risk of developing dementia, independent of vascular risk factors, changes in total brain volume, extent of white matter hyperintensities, and presence of covert infarcts (Romero et al., 2022). Iliff and colleagues found significantly reduced (~25%–60%) CSF influx in global AQP4 knockout mice relative to wildtype animals, which supports the premise that AQP4 channels facilitate fluid movement between the perivascular and interstitial spaces (Iliff et al., 2012). A recent report suggests that stagnation of interstitial fluid and increased interstitial volume may underlie the reduced glymphatic transport in AQP4 knockout mice (Gomolka et al., 2023). In an optimized hematoma expansion model, AQP4 knock-out resulted in an increased hematoma volume and increased severity of BBB breakdown, which indicates that AQP4 plays a role in attenuating hematoma expansion and maintenance of BBB integrity (Chu et al., 2020). In our prior work, we found that the expression of AQP4 is decreased in MMI rats which may contribute in-part to MMI induced glymphatic dysfunction (Venkat et al., 2017; Yu et al., 2019a). In the present study, we showed that MMI induces significant dilation of the PVS and reduces AQP4 expression around blood vessels compared to Sham rats, and AV-001 treatment significantly reduces PVS dilation and increases perivascular AQP4 expression compared to MMI rats.

PVS dilation and reduced AQP4 expression have been observed in VaD and are thought to contribute to glymphatic dysfunction (Venkat et al., 2017; Wang et al., 2017; Venkat et al., 2020). The glymphatic system is a glial-dependent, brain-wide, waste clearance pathway that facilitates the clearance of soluble proteins, neurotoxins and metabolic waste from the brain (Zhang et al., 2019). The glymphatic system also plays an important role in the delivery of nutrients and other essential molecules to the brain. Glymphatic dysfunction has been reported in several diseases including AD, stroke, diabetes mellitus, and VaD (Jiang et al., 2017; Venkat et al., 2017; Wang et al., 2017; Yu et al., 2019a; Lin et al., 2020). Multiple microinfarcts in the brain significantly impairs the global influx of CSF along the paravascular channels and increases the risk of amyloid plaque formation (Venkat et al., 2017; Wang et al., 2017). Impairment of the glymphatic pathway can lead to the aggregation of soluble proteins and neurotoxins which aggravate neuroinflammation and neurodegeneration and thus, dementia (Wang et al., 2017). In current study, our results indicate that MMI induces significant glymphatic dysfunction compared to Sham rats at 14 days after MMI, and AV-001 treatment significantly improves glymphatic function compared to non-treated MMI rats.

The relationship between inflammation and cognitive dysfunction has been well established, with increased levels of pro-inflammatory cytokines such as IL-1β, IL-6, C-reactive protein, and TNF-α found in both plasma and CSF of VaD patients (Tarkowski et al., 1999; Sjögren et al., 2004; Wada-Isoe et al., 2004; Yaffe et al., 2004; Zuliani et al., 2007). Chronic low-grade inflammation and neuroinflammatory factors have also been linked to age-associated morbidity and mortality, white matter injury, and cognitive impairment (Franceschi, 2007). Early and sustained inflammation in the brain can also cause white matter injury and cognitive impairment (Rosenberg, 2009; Wang L.-W. et al., 2012; Ben-Ari et al., 2019). In a transgenic mouse model of chronic neuroinflammation, overexpression of IL-6 in the brain was found to increase microglial proliferation and TNF-α expression (Gyengesi et al., 2019). MMI induces microglial and astroglial activation adjacent to sites of microinfarction at 3 days after MMI (Rapp et al., 2008b; Wang M. et al., 2012). Neuroinflammatory factors (such as TNF-α) secreted by reactive glial cells damage oligodendrocytes and worsen demyelination and white matter degeneration in VaD (Tarkowski et al., 2003; Solito and Sastre, 2012; Acosta et al., 2017). CXCL9 is a chemokine that participates in Th1-type immune responses, involving recruitment of effector T cells to sites of inflammation (Park et al., 2002). While CXCL9 is the most strongly dependent on interferon gamma for expression, TNF-α can induce CXCL9 mRNA expression in endothelial cells (Murphy, 2003). Increased expression of CXCL9 in the serum and CSF has been associated with cognitive decline and is increased in AD brain (Galimberti et al., 2003; Elkind et al., 2021). CSF levels of TNF-α, CXCL9, and IL-6 are also elevated in other neuroinflammatory diseases such as multiple sclerosis (Lepennetier et al., 2019). Thus, elevated inflammatory factor expression may initiate the neuropathological pathways that contribute to the development of VaD. Our findings demonstrate that in middle-aged rats, MMI leads to a significant increase in the expression of inflammatory factors such as TNF-α and CXCL9 in the CSF, as well as TNF-α, CXCL9, TLR-4 and IL-6 in the brain, whereas treatment with AV-001 significantly reduces the expression of these inflammatory factors. This reduction in inflammatory factors observed in the CSF and brain of middle-aged rats treated with AV-001 may have a positive impact on white matter integrity and cognitive outcome.

In addition to the increased risk for cognitive dysfunction associated with elevated inflammation, thrombin and PAI-1 have also been implicated in the development of VaD and AD. Thrombin is involved in fibrin formation and platelet aggregation in response to vascular injury, and its expression is increased in brain microvessels in AD patients (Grammas et al., 2006). In patients with VaD, plasma prothrombin levels were increased and systemic thrombin inhibition has been shown to improve cerebral metabolism in patients with silent cerebrovascular disease (Kario et al., 1996, 1999). PAI-1 plays a critical role in regulating the balance between thrombosis and fibrinolysis, and increasing PAI-1 levels can increase cardiovascular events via increasing thrombosis (Sillen and Declerck, 2020). Elevated PAI-1 levels in CSF may serve as a non-specific marker of neurological disease, and CSF PAI-1 levels were significantly increased in patients with AD, cerebral ischemia, central nervous system (CNS) infection, alcohol withdrawal seizures, and CNS neoplasia compared to patients without CNS disease (Sutton et al., 1994). PAI-1 is also the main physiological inhibitor of tissue plasminogen activator (tPA) and our prior work has shown that young adult, and middle-aged tPA−/− mice exhibit significant cognitive impairment, neuroinflammation, white matter injury, blood brain barrier leakage, glymphatic dysfunction, and increased deposition of thrombin, amyloid precursor protein, amyloid beta, and fibrin in the brain (Yu et al., 2019b). Therefore, targeting thrombin and PAI-1 may be a promising therapeutic strategy for VaD. Our data show that MMI significantly increases, while AV-001 treatment significantly decreases the expression PAI-1 in the CSF and brain and thrombin in the brain tissue of middle-aged rats. Of interest Vasculotide has been shown to counteract thrombin or lipopolysaccharide stimulated increases in trans-endothelial permeability *in vitro* (David et al., 2011; Wu et al., 2015). Inflammation is closely linked to vascular and endothelial dysfunction, as chronic inflammation can lead to endothelial dysfunction and subsequent vascular damage. Vascular and endothelial dysfunction have been implicated in VaD pathophysiology as evidenced by their association with impaired cognitive function and white matter hyperintensity volume (Hoth et al., 2007). A key role of endostatin in angiogenesis is preventing endothelial cell multiplication, migration, binding, and survival (Dhanabal et al., 1999; Dixelius et al., 2000). Endostatin, but not vascular endothelial growth factor was found to mediate a relationship between endothelial function and cognitive performance in patients with coronary artery disease (Isaacs-Trepanier et al., 2020). Activated endothelial cells up-regulate the expression of adhesion molecules and selectins. P-selectin is an adhesion molecule produced by activated platelets and endothelial cells which enhances procoagulant and proinflammatory activity (Kisucka et al., 2009). Elevated P-selectin is related to white matter hyperintensities in VaD patients with lacunar infarcts (Staszewski et al., 2018; Cipollini et al., 2019). Thus, addressing vascular and endothelial dysfunction could be a potential therapeutic strategy for preventing and treating MMI induced VaD and our data show that MMI significantly increases, while AV-001 treatment significantly decreases the expression of P-selectin in the CSF as well as endostatin in CSF and brain tissue.

Since cognitive function deteriorates with advancing age, one of the main imitations of this study is employing middle-aged rats to test the effectiveness of AV-001 treatment in MMI induced VaD. In our previous study, we evaluated the MMI model in young, middle-aged, and aged rats and found that the MMI model induces significant cognitive deficits in middle-aged and aged rats but not in young rats (Rapp et al., 2008b; Venkat et al., 2017, 2019). Aged rats also exhibited aged-induced cognitive impairment and MMI induced neurological deficits which can potentially interfere with cognitive tests (Rapp et al., 2008b; Venkat et al., 2017, 2019). Further investigation of the therapeutic effects of AV-001 in aged male and female rats subject to VaD are warranted.

## Conclusion

In this study, we demonstrate that treatment of MMI with AV-001 significantly reduces PVS dilation and increases perivascular AQP4 expression which may contribute to improved glymphatic function compared to MMI rats. AV-001 treatment also significantly reduces inflammatory factor expression in the CSF and brain which may contribute in-part to AV-001 treatment induced improvement in cognitive function.

## Data availability statement

The original contributions presented in the study are included in the article/supplementary material, further inquiries can be directed to the corresponding author.

## Ethics statement

The animal study was reviewed and approved by the Institutional Animal Care and Use Committee (IACUC) of Henry Ford Health.

## Author contributions

HG performed experiments and wrote sections of the manuscript. EF, LC, and AZ performed experiments. BP performed behavioral testing. JL-W performed immunostaining. TW and ML performed the statistical analysis. MC and PV contributed to the conception and design, supervision of the study and writing of the manuscript. All authors contributed to the article and approved the submitted version.

## Funding

This research was supported by the National Institute on Aging R01AG063750 (PV).

## Conflict of interest

The authors declare that the research was conducted in the absence of any commercial or financial relationships that could be construed as a potential conflict of interest.

## Publisher’s note

All claims expressed in this article are solely those of the authors and do not necessarily represent those of their affiliated organizations, or those of the publisher, the editors and the reviewers. Any product that may be evaluated in this article, or claim that may be made by its manufacturer, is not guaranteed or endorsed by the publisher.

## Abbreviations

AD: Alzheimer’s disease
Angpt-1: Angiopoietin-1
AQP4: Aquaporin-4
CCA: Common carotid artery
CNS: Central nervous system
CSF: Cerebrospinal fluid
cSVD: Cerebral small vessel disease
CXCL9: Chemokine ligand 9
ECA: External carotid artery
H&E: Hematoxylin and eosin
ICA: Internal carotid artery
IL-6: Interleukin-6
MMI: Multiple microinfarction
PAI-1: Plasminogen activator inhibitor-1
PVS: Perivascular space
RT-PCR: Real time polymerase chain reaction assay
TNF-α: Tumor necrosis factor-α
tPA: Tissue plasminogen activator
VaD: Vascular dementia
